# A randomized controlled trial on the effects of oxymetazoline nasal spray after dacryocystorhinostomy among adult patients

**DOI:** 10.1186/s13104-020-05076-4

**Published:** 2020-05-01

**Authors:** George Michael N. Sosuan, Felice Katrina T. Ranche, John Kenneth D. Lagunzad

**Affiliations:** Department of Ophthalmology and Visual Sciences, University of the Philippines Manila – Philippine General Hospital, Manila, Philippines

**Keywords:** Dacryocystorhinostomy, Oxymetazoline, Fistula, Congestion, Pain, Epistaxis

## Abstract

**Objectives:**

The study aimed to determine the effect of oxymetazoline nasal spray on the patency of the fistula created after dacryocystorhinostomy, specifically: to compare the success of fistula formation with oxymetazoline versus placebo, and to compare the incidence of post-operative congestion, pain and bleeding with oxymetazoline versus placebo.

**Results:**

The study was a single-center, randomized controlled, triple-masked study involving the patients of the Plastic-Lacrimal service of a national university hospital. Block randomization was done. Dacryocystorhinostomy was performed by a single-masked surgeon. The intervention group used oxymetazoline. The placebo group used sodium chloride. The data were collected by another masked investigator. The study showed no significant difference in terms of congestion, pain and epistaxis between the two groups at day 2 post-operation. The patency, presence of silicone tube, granuloma formation, and presence of bleeding on both day 2 and day 16 post-operation had no difference between the two groups. This study doesn’t support the use of oxymetazoline nasal spray after DCR, since it does not decrease the symptoms of congestion, pain and epistaxis after DCR. Aside from being an additional expense for patients, it also does not affect fistula formation and success rate of the surgery.

*Trial registration* Australian New Zealand Clinical Trial Registry: ACTRN12619001394134, Date registered 10/11/2019, Retrospectively Registered.

## Introduction

Dacryocystorhinostomy (DCR) was first described by Addeo Toti in 1904 as a surgical remedy for nasolacrimal duct obstruction (NLDO) [1]. In the study of Ezra, comparing the mucosal fistula following external DCR at post-operative day 1, 2 weeks and 6 months, the results showed that contracture of the fistula occurs mainly during the first 2 weeks post-operatively [2]. Currently, no established standard post-operative care can be found. It is usually surgeon or institution dependent. Topical intranasal medications such as sodium chloride, oxymetazoline or fluticasone can be given [3].

Oxymetazoline has α-2 adrenergic activity at usual doses and α-1 adrenergic activity at higher concentrations, both of which result in vasoconstriction and decongestion of the nasal mucosa [4]. In post-DCR patients, its theoretical use is prevention of hemorrhage in the middle meatus, and it is used for up to 3 days. The review of the available literature yielded no data on post-operative use of oxymetazoline.

In our institution, oxymetazoline and fluticasone nasal sprays are included as post-operative medications. Oxymetazoline nasal spray is instilled twice a day, on the operated side for the first 3 days. It adds around 35 USD to the patient’s expenses. This study may reduce unnecessary costs to our patients and would provide a guide for standard post-operative care.

Currently, there is no standard post-operative protocol available after dacryocystorhinostomy (DCR). This study aimed to determine the effect of oxymetazoline nasal spray on the patency of the fistula created after external DCR, specifically to compare the success of fistula formation with oxymetazoline versus placebo, and the incidence of post-operative congestion, pain and bleeding versus placebo.

## Main text

### Methodology

The study was a single-center, randomized controlled, quadruple-masked study involving the patients of the Plastic-Lacrimal (PL) service of a national university hospital, a tertiary referral center, from May 2017 to May 2018. The study was approved by the University Research Ethics Board. Informed written consent was obtained from each study participant. This study adhered to CONSORT guidelines.

There were five investigators involved: Investigator 1 did the block randomization of the subjects and the analysis of data; Investigator 2 did the surgery (external DCR with silicone intubation); Investigator 3 did the endoscopic evaluation; Investigator 4 instilled the medications. Investigator 5 assigned the two interventions as interventions A and B. The patients, investigators, outcome assessor, and data analyst were masked.

The following patients were included in the study: adult patients (> 18 year/o) of sound mind who can consent for the study, diagnosed with primary acquired NLDO with clearance for external DCR under general anesthesia, and assessed with pre-operative nasal endoscopic evaluation as suitable by Investigator 3. The patients with the following characteristics were excluded from the study: previous lacrimal or nasal surgery; active inflammation of the lacrimal apparatus or nasal passages; known case of uncontrolled hypertension, diabetes or hyperthyroidism; use of monoamine oxidase inhibitor or tricyclic antidepressants; known allergy to oxymetazoline; or breastfeeding patients. For cases with bilateral obstruction, only one side was included in the study.

The measured outcomes were grouped in two categories—based on the patient’s symptoms of congestion, pain and epistaxis; and based on endoscopic findings of patency, presence of silicone tube in the fistula, granuloma formation and bleeding. The primary outcome measure was patency, which was used as the basis of our sample size computation. The patency was evaluated during nasal endoscopy by the egress of fluorescein-stained (yellow-stained) fluid from the fistula while doing lacrimal apparatus irrigation.

At the start of the study, Investigator 5 wrapped bottles of 0.05% oxymetazoline nasal spray and 0.65% sodium chloride nasal spray which served as our placebo and stored them in two separate boxes. Investigator 5 decided which box would be intervention A, and which would be intervention B. This information was kept from the other investigators, and the bottles were stored in the Hospital Ward.

Twenty patients of the Plastic-Lacrimal (PL) service of a national university hospital, a tertiary referral center, from May 2017 to May 2018 were screened. Four patients were excluded because 3 had active infection and 1 had uncontrolled hypertension. Sixteen patients met the criteria and were included in the study. Block randomization was done to assign each patient equally to either Intervention A or Intervention B (Additional file 1: Appendix A) and concealment using numbered containers was done by Investigator 1.

External DCR with silicone intubation was performed by Investigator 2. A standard operative technique was followed for all the patients. The patients were under general anesthesia, positioned supine on the table. Subcutaneous 2% lidocaine + 1/100,000 epinephrine was injected along the side of the nose adjacent to the medial canthus. The middle turbinate was packed with gauze strips soaked with 0.05% oxymetazoline. A curvilinear incision about 10 mm in length was made, starting between the medial canthus and the nasal bridge, and directed inferiorly tangential to the nasal ala. The dissection was carried to the periosteum of the anterior lacrimal crest, which was incised and lifted to expose the lacrimal sac fossa. An osteotomy measuring approximately 10 × 10 mm was created using Kerrison rongeurs. The nasal pack was then removed, and the exposed nasal mucosa was infiltrated with 2% lidocaine + 1/100,000 epinephrine. U-shaped flaps were created on the lacrimal sac and the nasal mucosa. A bicanalicular silicone tube (FCI Ophthalmics) was inserted via the upper and lower puncta, threaded down to the middle meatus, and secured with one square knot. The flaps were anastomosed with 2 simple interrupted sutures. The orbicularis was closed using 5 simple interrupted sutures, and the skin was closed using 5 simple interrupted sutures. Vicryl 6-0 was used for all sutures.

Coordination with the anesthesiologists was done to ensure the use the same medications for immediate postoperative analgesia. The medications included paracetamol 600 mg/IV every 6 h for 3 doses and ketorolac 30 mg/IV every 6 h for 2 doses, then paracetamol 500 mg every 6 h as needed for pain. Post-operative monitoring done in the ward included blood pressure, heart rate and episodes of epistaxis. The intervention group used 0.05% oxymetazoline nasal spray post-operatively, instilled twice a day (8AM and 8PM), on the operated side for the first 3 days; on the other hand the placebo group used 0.65% NaCl nasal spray post-operatively, instilled the same way as the intervention group. The patients were admitted at the Hospital Ward, and the medications were administered by Investigator 4. The patients were followed-up on post-operative day 2 for assessment of symptoms and endoscopy, and on post-operative day 16 for repeat endoscopy by Investigator 3 (Additional file 1: Appendix B).

The endoscopy machine of the hospital was used for the study. The specifications of the endoscopy machine are as follows: the light source is from Welch Allyn Solarc with Storz fiber-optic rigid nasal endoscope, size 5.0 mm, tip angled at 30 degrees, length of 24 cm.

The data collected by Investigator 3 were: symptoms of congestion, pain and epistaxis on day 2, and endoscopic findings of patency, presence of tube, granuloma formation and bleeding on day 2 and day 16. These data were analyzed by Investigator 1. After data analysis was completed, Investigator 5 revealed that the intervention A was 0.65% NaCl nasal spray, and the intervention B was 0.05% oxymetazoline nasal spray. The success rate of DCR was assessed by checking the patency of the fistula formed by lacrimal irrigation apparatus at 6 months post-operation.

The sample population was computed using the Open Source Statistics for Public Health Software (Additional file 1: Appendix C). The sample size was based on the primary outcome of patency. The rate of patency in external DCR usually ranges from 80 to 90%, so an average of 85% is chosen, and pegged the arbitrary risk difference to be 80%. After plugging the data into Open Source Statistics, the computed total study population is 14 with 95% confidence interval and 80% power. A minimum of 7 participants per intervention was needed for the study. Intention-to-treat analysis was used (Additional file 2).

Descriptive statistics were used to analyze the data. The data were encoded in Microsoft Excel and then cleaned and analyzed using R 3.5.1. The analysis was blinded to treatment received by the two groups. Fisher exact statistics was used for comparison of groups in terms of categorical variables. For the age, unpaired *t* test was used. For the number of comorbidities, Kruskal–Wallis Rank sum test was used due to non-normality of data.

### Results

Sixteen participants were included in the study. Two participants dropped out. One was due to a scheduling conflict on the day of surgery, and the other one was due to inability to do endoscopic examination post-operatively.

There were no significant differences in terms of age, sex, and number and type of co-morbidities between the two groups (Table 1).

**Table 1:** Demographic and clinical characteristics of trial participants

Variable	NaCl group	Oxymetazoline group	P-value
Age (mean (SD))	54.12 (17.80)	61.25 (12.95)	0.375
Sex (%)	1 (12.5)	1 (12.5)	1.000
No. of Comorbids (median [IQR])	0.50 [0.00, 2.25]	1.00 [0.00, 1.25]	1.000
Co-morbidities			
HTN (%)	4 (50.0)	4 (50.0)	1.000
Dyslipidemia (%)	3 (37.5)	1 (12.5)	0.569
DM/IFG (%)	2 (28.6)	2 (28.6)	1.000

*HTN* hypertension, *DM* diabetes mellitus, *IFG* impaired fasting glucose

**Fig. 1:**
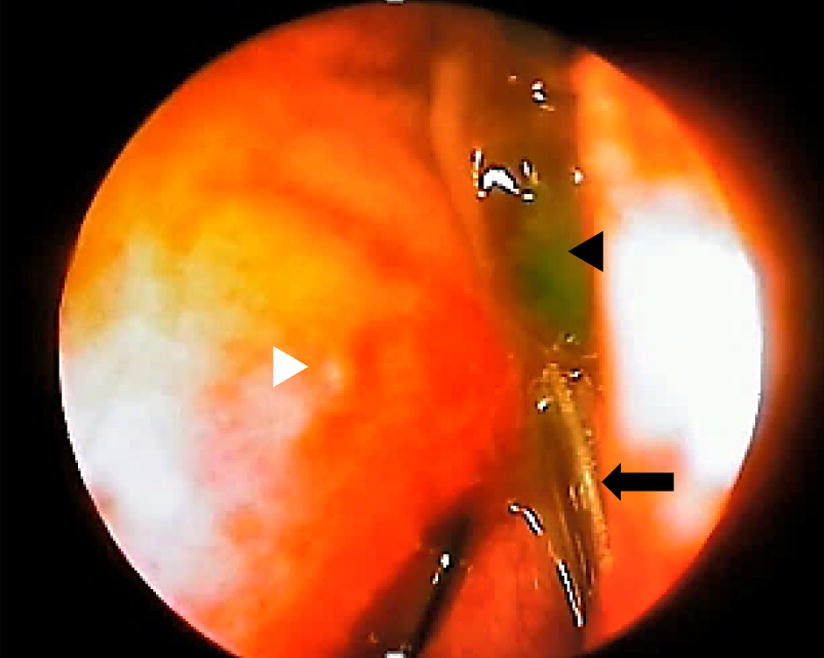
The nasal endoscopy at day 16 showing the silicone tube (arrow) being in place near the middle meatus and inferior turbinate (white arrowhead), the absence of bleeding, the absence of granuloma formation, and the patency of the fistula as confirmed by egress of fluorescein-stained fluid (black arrowhead)from the fistula during lacrimal apparatus irrigation

The primary outcome of this study was patency. The endoscopic evaluation assessed the patency of the fistula, tubes being in place, presence of granuloma formation, and presence of bleeding (Fig. 1). There was no difference in the patency of both interventions. One participant in each group had a failed operation because of infection. The one which failed in the NaCl group had dacryocystitis at 3 months post-operation, while the one which failed in the oxymetazoline group had dacryocystitis at 1-month post-operation. There was no significant difference in terms of congestion, pain and epistaxis between the two groups at day 2 post-operation, although slightly more participants in the oxymetazoline group experienced congestion and epistaxis (Table 2). The patency, tubes being in place, presence of granuloma formation, and presence of bleeding on both day 2 and day 16 post-operation had no difference (Table 2).

**Table 2:** Outcomes according to treatment

Variable	NaCl group	Oxymetazoline group	P-value
Congestion (%)	2 (25.0)	3 (37.5)	1.000
Pain (%)	1 (12.5)	1 (12.5)	1.000
Epistaxis (%)	2 (25.0)	4 (50.0)	0.608
Success (%)	6 (75.0.)	6 (75.0)	1.000
Day 2 outcomes			
Patency (%)	7 (87.5)	7 (87.5)	1.000
Tube in Place (%)	7 (87.5)	7 (87.5)	1.000
Granuloma formed (%)	1 (12.5)	1 (12.5)	1.000
With bleeding (%)	1 (12.5)	1 (12.5)	1.000
Day 16 outcomes			
Patency (%)	7 (87.5)	7 (87.5)	1.000
Tube in place (%)	7 (87.5)	7 (87.5)	1.000
Granuloma formed (%)	1 (12.5)	1 (12.5)	1.000
With bleeding (%)	1 (12.5)	1 (12.5)	1.000

### Discussion

The success rate in this study was a little lower than the published success rates in the literature. The known success rates of external DCR ranges from 80–90%. In our study, the success rate was around 75%. In the study of Dhar 2010, they noted that infection was one of the most common cause of failed DCR. The recurrence rate of dacryocystitis in their study was low at 12%. They noted the following as risk factors for recurrent infections as follows: colonization of unusual or resistant bacteria, distortion of bony anatomy and in rare cases neoplasms. In our study, the occurrence of dacryocystitis after DCR was around 14%, which was similar to the published literature [5].

Oxymetazoline nasal spray is administered to alleviate discomfort and to improve the visualization during nasal endoscopy [6]. It is used pre-operatively in the operating area in endoscopic sinus surgery and dacryocystorhinostomy to promote decongestion and vasoconstriction and minimize nasal mucosa bleeding during surgery [7, 8]. Oxymetazoline nasal spray is found to be non-inferior to cocaine and epinephrine in nasal surgeries with the benefit of less systemic complications of arrhythmia, hypertension and cardiac arrest [8]. The benefits of oxymetazoline nasal spray when given post-operatively after nasal septoplasty include reduced bleeding and crust formation in the early post-operative period [9]. In our study, the benefit of adding oxymetazoline nasal spray post-operatively after DCR to decrease congestion and pain and minimize bleeding was not demonstrated as compared to 0.65% NaCl nasal spray. Although not statistically significant, an increase in the occurrence of congestion and epistaxis was noted in this study with use of oxymetazoline.

The addition of post-operative oxymetazoline nasal spray did not alter the fistula formation at 2 weeks as well as the success rate as compared to 0.65% NaCl nasal spray in our study. This study shows that there is no benefit in using oxymetazoline nasal spray after dacryocystorhinostomy to decrease congestion, pain and epistaxis. The addition of oxymetazoline also has no effect on fistula formation at 2 weeks and in the patency rate at 6 months.

## Limitations

The limitations of the study include the unavailability of a true placebo for commercially available 0.05% oxymetazoline nasal sprays, which should consist of the chemical formulation of the commercially available 0.05% oxymetazoline nasal spray without the active ingredient. Due to the unavailability, 0.65% saline solution was used as the placebo/control. Secondly, the endoscopic evaluation was only until the second post-operative week. In practice, silicone tubes can be maintained for 3 to 6 months. Two weeks post-operative endoscopic evaluation was chosen because of the length and variability of the follow-up period and because contracture of the mucosal fistula occurs mainly in the first 2 weeks after surgery. Furthermore, because both the intervention and placebo are only instilled in the first 3 days after surgery, they are no longer expected to have effect on the surgical site beyond the second postoperative week. This was a pilot study on the effects of oxymetazoline nasal spray after dacryocystorhinostomy. The authors recommend further studies to increase the sample size of the population. Further studies may also focus on the comparison of fluticasone nasal spray versus placebo after dacryocystorhinostomy.

## Supplementary information

**Additional file 1. **Appendix A (Sample Block Randomization), Sample B (Protocol Flowchart), Protocol C (Sample Size Calculation.

**Additional file 2.** Tabulated Data Set.

## Data Availability

The datasets used and analyzed in the current study are available from the corresponding author on reasonable request.

## Abbreviations

DCR: Dacryocystorhinostomy
NLDO: Nasolacrimal duct obstruction
USD: United States Dollar
PL: Plastic-lacrimal
