# Supplementary material for: A randomized controlled trial on the effects of oxymetazoline nasal spray after dacryocystorhinostomy among adult patients
Source: BMC Res Notes. 2020 May 1;13:236. doi: 10.1186/s13104-020-05076-4 (PMC7195701; doi:10.1186/s13104-020-05076-4)
Supplement: Supplementary file 1 — Additional file 1. Appendix A (Sample Block Randomization), Sample B (Protocol Flowchart), Protocol C (Sample Size Calculation. [file 13104_2020_5076_MOESM1_ESM.docx]

**Appendix A**

**Sample Block Randomization**


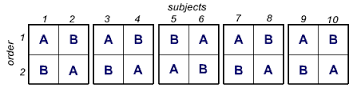


Block randomization was done to assign each patient equally to either Intervention A or Intervention B (See Appendix A) by Investigator 1.

**Appendix B**

**Protocol Flowchart**

Investigator 5 assigned medication into intervention A and B.

Data Analysis (Investigator 1)

Assessment of symptoms at Day 2 Nasal endoscopy at Day 2 and Day 16 (Investigator 3)

0.05% Oxymetazoline nasal spray x 3 days (Investigator 4)

0.65% NaCl nasal spray x

3 days (Investigator 4)

Block Randomization

(Investigator 1)

External DCR

(Investigator 2)

Plastic Lacrimal Clinic

May 2017 to May 2018

Recruitment

(Investigator 1)

16 participants

Inclusion and Exclusion Criteria

**Appendix C**

**Sample Size Calculation**

|  | | | | |
| --- | --- | --- | --- | --- |
| **Sample Size:X-Sectional, Cohort, & Randomized Clinical Trials** | | | | |
| Two-sided significance level(1-alpha): | | | 95 |  |
| Power(1-beta, % chance of detecting): | | | 80 |  |
| Ratio of sample size, Unexposed/Exposed: | | | 1 |  |
| Percent of Unexposed with Outcome: | | | 5 |  |
| Percent of Exposed with Outcome: | | | 85 |  |
| Odds Ratio: | | | 110 |  |
| Risk/Prevalence Ratio: | | | 17 |  |
| Risk/Prevalence difference: | | | 80 |  |
|  | | | | |
|  | **Kelsey** | **Fleiss** | **Fleiss with CC** |  |
|  | | | | |
| Sample Size - Exposed | 7 | 5 | 7 |  |
| Sample Size-Nonexposed | 7 | 5 | 7 |  |
|  | | | | |
| Total sample size: | 14 | 10 | 14 |  |
|  | | | | |
| References | | | | |
| Kelsey et al., Methods in Observational Epidemiology 2nd Edition, Table 12-15 | | | | |
| Fleiss, Statistical Methods for Rates and Proportions, formulas 3.18 &3.19 | | | | |
| CC = continuity correction | | | | |
| Results are rounded up to the nearest integer. | | | | |
| Print from the browser menu or select, copy, and paste to other programs. | | | | |

Results from OpenEpi, Version 3, open source calculator--SSCohort 
Print from the browser with ctrl-P 
or select text to copy and paste to other programs.

From: http://www.openepi.com/SampleSize/SSCC.htm
